# A framework for multi-faceted content analysis of social media chatter regarding non-medical use of prescription medications

**DOI:** 10.1186/s44247-023-00029-w

**Published:** 2023-08-07

**Authors:** Shaina Raza, Brian Schwartz, Sahithi Lakamana, Yao Ge, Abeed Sarker

**Affiliations:** 1 Dalla Lana School of Public Health, University of Toronto, Toronto, ON, Canada.; 2 Vector Institute for Artificial Intelligence, Toronto, ON, Canada.; 3 Department of Biomedical Informatics, School of Medicine, Emory University, Atlanta, GA, USA.

**Keywords:** Substance use, Natural language processing, Machine learning, Social media

## Abstract

**Background:**

Substance use, including the non-medical use of prescription medications, is a global health problem resulting in hundreds of thousands of overdose deaths and other health problems. Social media has emerged as a potent source of information for studying substance use-related behaviours and their consequences. Mining large-scale social media data on the topic requires the development of natural language processing (NLP) and machine learning frameworks customized for this problem. Our objective in this research is to develop a framework for conducting a content analysis of Twitter chatter about the non-medical use of a set of prescription medications.

**Methods:**

We collected Twitter data for four medications—fentanyl and morphine (opioids), alprazolam (benzodiazepine), and Adderall^®^ (stimulant), and identified posts that indicated non-medical use using an automatic machine learning classifier. In our NLP framework, we applied supervised named entity recognition (NER) to identify other substances mentioned, symptoms, and adverse events. We applied unsupervised topic modelling to identify latent topics associated with the chatter for each medication.

**Results:**

The quantitative analysis demonstrated the performance of the proposed NER approach in identifying substance-related entities from data with a high degree of accuracy compared to the baseline methods. The performance evaluation of the topic modelling was also notable. The qualitative analysis revealed knowledge about the use, non-medical use, and side effects of these medications in individuals and communities.

**Conclusions:**

NLP-based analyses of Twitter chatter associated with prescription medications belonging to different categories provide multi-faceted insights about their use and consequences. Our developed framework can be applied to chatter about other substances. Further research can validate the predictive value of this information on the prevention, assessment, and management of these disorders.

## Background

Substance use and its consequences are a major global health problem. In the United States, for example, more than 100,000 deaths were reported in the 12 months leading up to July 2022 [1]. Researchers and public health professionals across the globe are finding it challenging to keep up with emerging trends in how consumers use different psychoactive substances (e.g., alcohol, morphine, nicotine, and certain pain medications). Overdoses can result from both prescription medications and illicit substances. According to a 2015 survey conducted by the Substance Abuse and Mental Health Services Administration (SAMHSA) [2], 18.9 million Americans aged 12 and older misused prescription drugs in the previous year. Though the organizations, such as the Centers for Disease Control and Prevention (CDC) [3], United States Food and Drug Administration (FDA) [4], and Drug Information Portal (DIP) [5] frequently publish updates on the latest substances and their usage trends, such information often contains delays. Traditional sources also lack the information about patient experiences and behavioural trends associated with the substances. Social media serves as a potentially high-utility source of information that can be obtained in close to real-time [6]. However, information on social media is noisy and in free text format, and extracting such information requires the development of natural language processing (NLP) and data-centric methods.

Social media platforms like Twitter enable the identification and tracking of emerging substance use trends, including related terms, indicators, and effects [7]. A review of Twitter data [8] revealed numerous mentions of opioid terms such as fentanyl, heroin, and morphine in tweets. As reported by numerous past studies [9], many social media subscribers openly discuss their substance use with their online networks, even if they might not feel comfortable discussing these topics with their doctors. Recent studies [10–12] also suggest the potential to use social media data to supplement survey results in studying psychoactive substances and their effects. The primary advantage of social media lies in the rapid dissemination of information from the data. In this study, we build upon existing research in this area and develop an NLP framework to extract multifaceted information about substances prone to non-medical use.

We focus this study on non-medical use of prescription medications belonging to the categories of opioids, benzodiazepines, and stimulants. Based on their popularity as some of the most addictive and/or commonly used prescription medications in the market [13], we include the drugs morphine, fentanyl, Adderall^®^ (amphetamine mixed salts), and alprazolam, and extract information on these drugs from a social media corpus (Twitter). Alprazolam [14] is a benzodiazepine used to treat anxiety disorders; fentanyl [15] is a strong synthetic opiate to treat cancer patients’ pain, and much of the illicit opioid supply in the United States is now contaminated with fentanyl [16, 17]; Adderall^®^ [18] is a stimulant used to treat Attention Deficit Hyperactivity Disorder (ADHD); and morphine [19] is an opiate that when prescribed by a doctor is used to treat pain. Due to the known non-medical use of these prescription medications, it is important, to gain insights into patterns of use, risk factors, and behaviours associated with substance non-medical use.

The primary goal of this research is to develop a programming framework that gathers multifaceted insights about the non-medical use of prescription medications known to be addictive. The specific contributions of this work are:

An NLP framework is proposed that integrates multiple components, such as a preprocessor to process the social media data, a named entity recognition (NER) model for identifying and categorizing key information (named entities) related to medications in the texts, and a topic modeler for identifying and clustering latent concepts is proposed. These components are stacked together in a pipeline structure to produce outputs for studying substance use.

A two-mode evaluation scheme is presented, consisting of a quantitative analysis for the performance comparison of several baseline methods for the NER task, and a qualitative analysis to demonstrate the effectiveness of the proposed approach, including a discussion of its advantages and limitations.

By capturing individual experiences, behaviors, and perspectives from user-generated content, the proposed approach bridges the gap between traditional sources and real-world experiences. This enriches our understanding of substance use and its impact on individuals and communities, offering a holistic view of the issue and facilitating more informed decision-making in addressing the problem.

### Previous works

The intersection of NLP and the study of drugs and medications has attracted increasing research interest in recent years. One line of research [20–25], in this regard, explored the application of NLP, in particular relation extraction and NER techniques to identify and analyze drugs and medicine related information. These works primarily utilize data from scientific literature [26] or clinical research studies [21] to extract crucial insights pertaining to the subject matter. Another line of research [9, 27–29] employed machine learning algorithms to classify social media data, such as tweets [9] or Reddit posts [30], and determine patterns of drug misuse, providing valuable insights into the public perception and understanding of the issue. Through diverse NLP techniques, these studies were able to extract and analyse textual data, uncovering trends and common themes associated with drug misuse within these virtual communities.

In one study [31], an NLP-based system was developed to monitor and detect potential instances of prescription drug misuse on social media platforms. The authors demonstrated the effectiveness of their approach in identifying and flagging content that may indicate drug misuse, highlighting the potential of NLP in supporting public health surveillance efforts. Another study [32] used sentiment analysis to predict users’ opinions on prescription medications based on their social media content. A separate investigation [33] explored the role of sentiment analysis in understanding public opinions about the non- medical use of prescription drugs. Another related study [34] analysed the language used in tweets to understand patterns of prescription drug misuse. Taking a different approach, researchers in [35] developed an NLP system to automatically detect adverse drug reactions from social media data.

These studies demonstrate the growing interest in leveraging NLP techniques to study the non-medical use of prescription medicines and related issues. By building on the findings of the previous works [20, 24, 36–39], our research aims to further advance the understanding of the phenomenon of non-medical use of prescription drugs through NLP. Distinct from prior research, we present an NLP pipeline that incorporates various components, such as NER, topic modelling, and evaluation methods. This comprehensive approach aims to provide a holistic understanding of the complex phenomenon.

## Methods

### Data

The data comprises tweets and was collected via the Twitter academic Application Programming Interface (API). All tweets mentioned at least one of the four previously mentioned prescription medications (alprazolam, fentanyl, morphine, and Adderall^®^), which were selected in consultation with a toxicology expert. A total of approximately 2 million tweets were collected in this process. Data were collected using the medication generic names, trade names, and their common misspellings [40]. Since most of the chatter on Twitter does not represent non-medical use, a state-of-the-art supervised classification model that fuses multiple machine learning methods (BiSLTM and BERT-based methods) using a logistic regression [41] was applied to only keep posts that represented non-medical use or personal consumption.

From this continuously-running pipeline that was developed in our prior works [40, 41], we took a sample of the Twitter dataset that we refer as our corpus. In our study, we used specific inclusion and exclusion criteria to ensure the quality and relevance of the tweets in the data collection process. The dataset consists of a five-month sample, covering the period from May 31, 2021, to October 31, 2021. During this time, we implemented the following rules:

#### Inclusion criteria:

1. Tweets must be in English.
2. Tweets must fall within the specified date range.
3. Tweets must mention at least 1 keyword (including spelling variants and trade names) for the included medications alprazolam, fentanyl, morphine, and Adderall^®^.
4. The tweets were classified using a fusion-based classifier [41] to indicate non-medical use or consumption, as well as self-reports of non-medical use by Twitter subscribers. This classifier is a supervised model that combines the probabilities of each tweet from base classifiers (BiLSTM, AlBERT, and RoBERTa) using a logistic regression classifier (metaclassifier).

#### Exclusion criteria:

1. Tweets containing spam, advertisements, or irrelevant content are excluded.
2. Tweets from accounts that were determined to be bots by the system described in Davoudi et al. [42] are excluded.
3. Tweets with less than a certain number of words or characters are excluded to ensure meaningful content.
4. Retweets and quoted tweets are excluded to avoid duplication and ensure originality.

Estimation of the demographic distribution of the subscribers included in this study has been reported in the prior publication by Yang et al. [12]. These estimations show that the demographics of the subscribers are very closely reflective of the demographics reported in the National Survey on Drug Use and Health (NSDUH) [43] in terms of race and gender, with age-group being an anomaly since younger people are overrepresented on Twitter. We were particularly cautious about not allowing our findings to be biased by information posted by bots, so all posts from suspicious accounts, as detected by the system proposed in Davoudi et al. [42] were excluded.

After applying this filtering process, 150k tweets were retained, which provided useful information about language patterns, topics, and keyword analysis related to the four prescription drugs. Our analysis was focused on a specific time and a specific set of drugs, and this much size of dataset provided sufficient information for our research goals.

### Proposed natural language processing framework

**Fig. 1:**
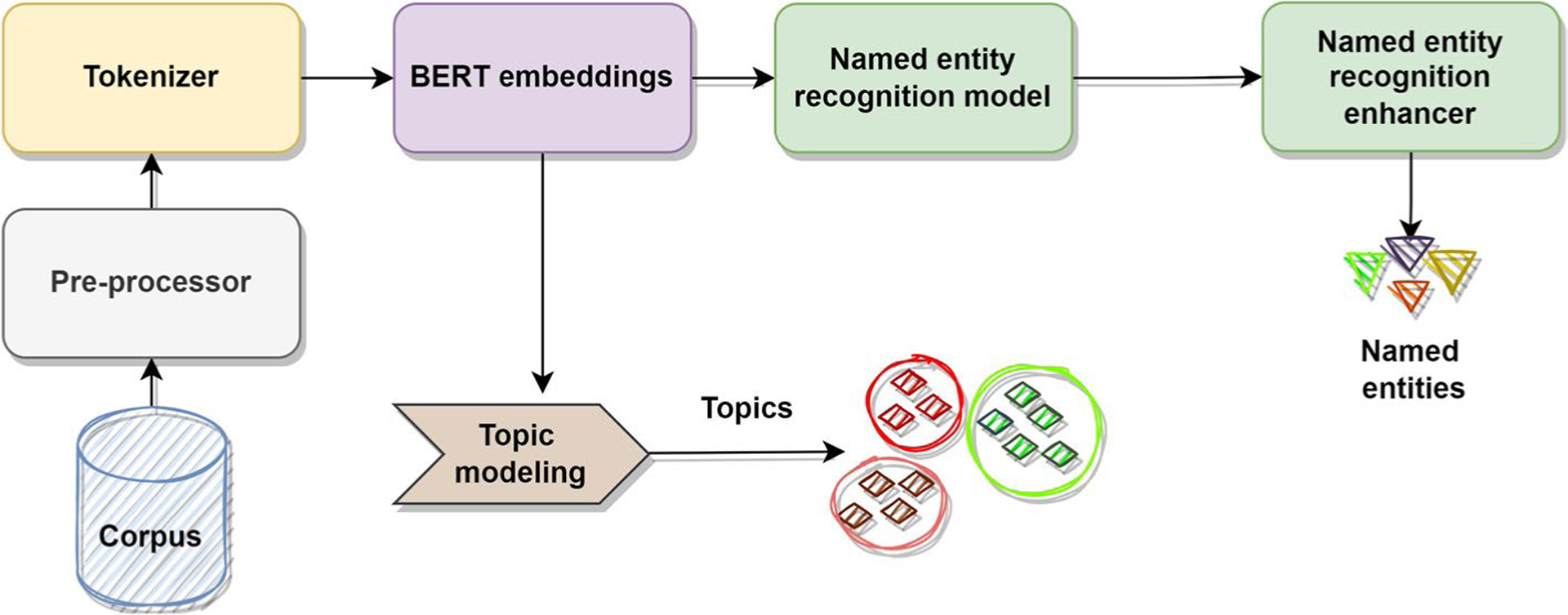
Overview of proposed NLP framework for extracting non-medical prescription medication use-related information from Twitter chatter

We developed an NLP framework (Fig. 1) that consists of a pre-processor, a tokenizer, a BERT embedding module, a named entity recognition (NER) model, a NER enhancer, and a topic modelling component. Each component was chosen based on its effectiveness in addressing specific NLP tasks, as demonstrated in previous research. For example, in NLP, a pre-processor is employed to prepare raw text data for further analysis [26]. BERT [44] -based models can be utilized to capture contextual representations from the data that improve the analysis. Named Entity Recognition (NER) can effectively extract information on named entities, such as people, ages, and locations, as well as clinical or medical entities including drugs [45, 46], from the text data. Topic modelling, which is a widely used NLP method, can uncover hidden patterns in the data, such as non-medical use of prescription medicines and related issues [47, 48]. The novelty of our approach lies in integrating different NLP modules in a pipeline structure. These insights can be valuable for developing targeted intervention strategies. We briefly describe each component of the proposed NLP framework below.

### Corpus

The dataset we used is described in the Data subsection above, is referred to as the corpus, and it comprises tweets. Each tweet is a row in the dataset, with columns for the tweet ID, user ID, tweet timestamp, tweet text, and the medication mentioned. We de-identified the user IDs to protect anonymity during the modelling phase.

### Pre-processor

The pre-processor module [49] is responsible for receiving textual data from the corpus and preparing it for further analysis. It reads the text of each record as either a string or an array and then cleans the data to remove any missing values, noisy data, or other irregularities that may affect the subsequent analysis. To identify sentence boundaries, it uses regular expressions (given in Appendix A, Table S1) that match common punctuation marks such as periods, exclamation points, and question marks. It also takes into account other features that might indicate the end of a sentence, such as multiple periods or ellipses. Once the pre-processor has identified the sentence boundaries, it converts the text into a format that the subsequent module of the system can comprehend. The pre-processor plays a critical role in ensuring the accuracy and reliability of the subsequent steps.

### Tokenizer

The tokenizer [49] is a module in our system that receives pre-processed data from the pre-processor as input. Its primary function involves dividing the input text into smaller chunks or tokens, such as words or phrases. This process is critical for downstream analysis because it allows the system to understand the meaning and structure of the text data.

The transformed data, which contains the tokens (words) corresponding to each record, is the tokenizer’s output. This transformed data is frequently represented as a matrix, with each row representing a record and each column representing a token.

### BERT embeddings

The BERT embeddings module in our framework leverages pre-trained NER models, specifically ner_jsl_biobert [50] and bert-clinical for adverse drug events [51] to extract features from tweets. These pre-trained models are taken from JohnSnowLabs [52]. The ner_jsl_biobert [50] model is a BERT-based model that is pre-trained on large-scale biomedical text corpora, making it ideal for identifying and classifying entities related to drugs and other medical terms. The bert-clinical model for adverse drug events [51] is another NER model that is specifically designed to identify and classify entities related to potential side effects, drug interactions, and other adverse events associated with medications.

We chose to use Bidirectional Encoder Representations from Transformers (BERT) [44]-based embeddings for this task due to their demonstrated effectiveness in various NLP task. Our embedding module is designed to be flexible, allowing for the integration of other pre-trained embeddings, such as GloVe [53], BERT-based variants, or other similar models, depending on the specific requirements of the analysis.

### Named Entity Recognition (NER)

**Fig. 2:**
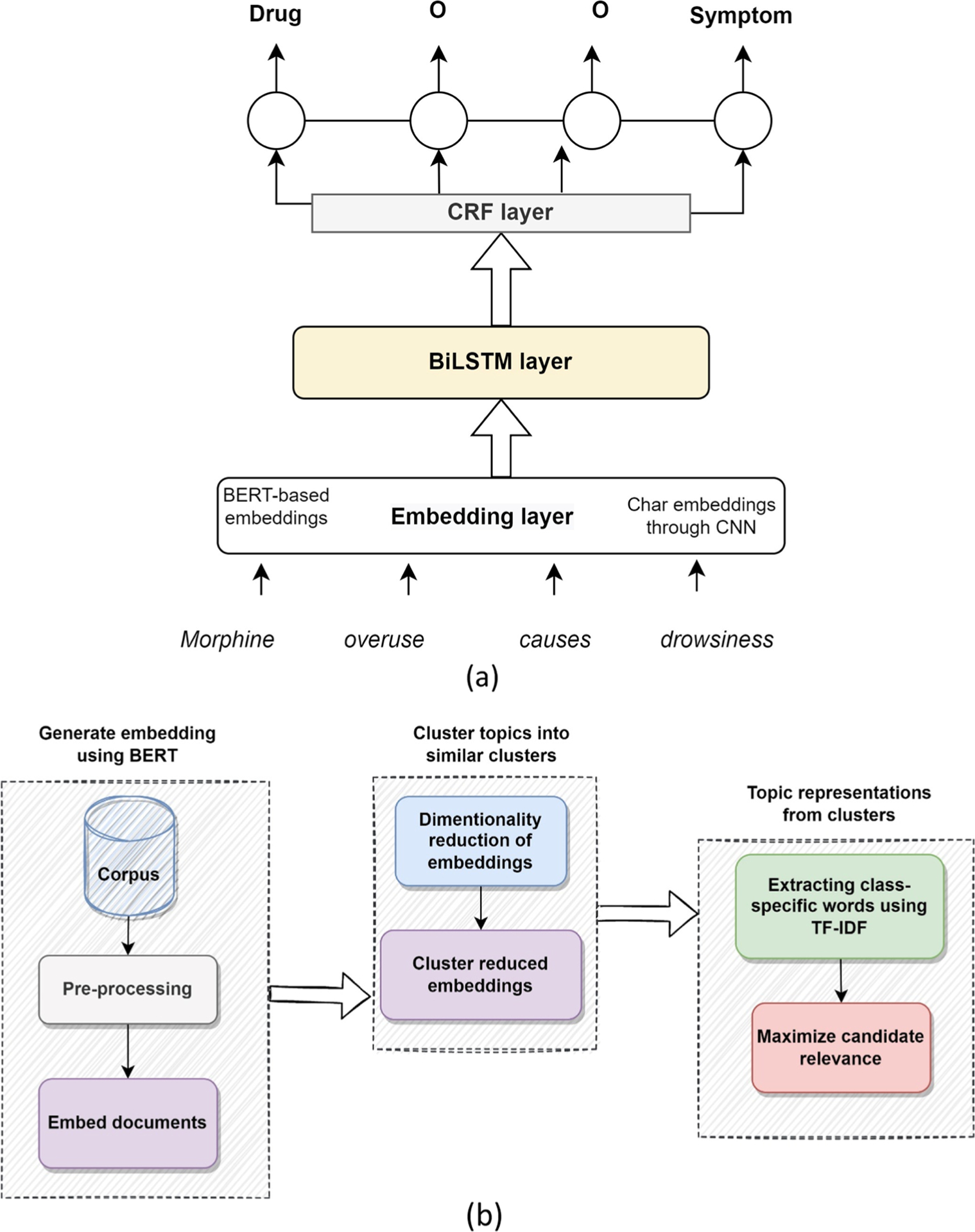
The main components of the proposed NLP framework (**a**) Named Entity Recognition architecture with embedding layer, BiLSTM layer and CRF layer, (**b**) Topic modelling component

NER [54] is the task of identifying and categorizing key information (such as a person, an organization, or an event) in text. The NER model used in this work (shown in Fig. 2a) is based on Bidirectional Long Short-Term Memory (BiLSTM)-Convolutional Neural Networks (CNN)—Conditional Random Field (CRF) model [55] with some customizations. Like the vanilla BiLSTM-CNN-CRF [55] model, we extract the character-level features from the word tokens via the CNN layer, but we also consider the contextualized embeddings for each token via pre-trained biomedical embeddings. The intuition is that the most useful features come from the contextualized embeddings [56] in addition to the character-level features.

The first layer in the model (Fig. 2a) is the embedding layer. We use the BERT-based embeddings (defined above) for word representation. We also apply the CNN to embed each character and get a vector representation. The second layer in the NER framework is the BiLSTM which takes as input the output vector from the embedding layer. This layer captures the context features to obtain more comprehensive semantic information from the texts. To ensure that the predicted labels are valid, the CRF layer captures the dependency relationship between the named tags and constrains them to the final predicted labels. The output of this model is the named entities. The named entities used in this work are taken from the JohnSnowLab pre-trained models [57] (ner_jsl_biobert and bert-clinical for adverse drug events) and are given in Appendix A, Table S2.

### Named entity recognition enhancer

The Inside, Outside, before (IOB) [58] format is a widely-used tagging scheme for named entities in NER tasks, as defined in the CoNLL-2003 shared task [59]. However, this format is designed for machine learning algorithms and NER training tasks. It can be difficult to comprehend it for use, as it uses tags like “B-”, “I-”, and “O” to represent the beginning, inside, and outside of named entities, respectively. This NER enhancer component converts the IOB representation of named entities to a user-friendly format. It also eliminated the entities with ‘O’ labels. The output of this annotator is referred to as the chunk. A chunk is a portion of a sentence that is tagged with named entities.

### Topic modelling

Topic modelling [60] is an unsupervised machine learning technique that can scan a collection of documents, detect word and phrase patterns within them, and automatically cluster words into groups based on similarity. We leverage the BERTopic [61] method and the outline of this technique is shown in Fig. 2b and explained next.

The first stage of topic modelling is to generate document (tweets) embeddings. After extracting and pre-processing, the embeddings are obtained from the BERT embedding. The second stage is to group the topics into clusters, where we reduce the dimensionality of the embeddings using Uniform Manifold Approximation and Projection [62] and then cluster using Hierarchical and Density Based Clustering [63] algorithm. The third stage is to find the topic representations from the clusters. The class-based Term frequency-inverse document frequency (c-TFIDF) [64] method is used to model the importance of words in clusters. This generates topic-word distributions for each cluster to create dense clusters. In the later steps, the IDF values are multiplied by the term frequency of documents at a timestep to model how topics change over time.

### Evaluation

In this paper, we adopted a two-mode evaluation strategy. First, we conducted a quantitative evaluation, and then a qualitative analysis.

For the quantitative analysis, we evaluated the performance of individual components, such as the NER model and the topic modelling, by comparing their results to ground truth data. Following the standard work in NLP evaluation [65], we use the metrics such as precision, recall, and F_1_-score to quantify the performance of these components. By performing a fivefold cross-validation, we were able to assess the consistency of our models across different data subsets, further demonstrating the robustness of our approach. We also assessed the effectiveness of the NLP framework through a qualitative evaluation. This involved obtaining a more in-depth understanding of how well the system performs in identifying relevant information and patterns related to the non-medical use of prescription medicines and related issues.

Our evaluation scheme can be categorized as a combination of summative and formative assessments. The summative evaluation [66] focuses on measuring the performance of the developed system and its components using numerical metrics after their implementation, while the formative evaluation [66] aims to provide a deeper understanding of the NLP models, their effectiveness and identify areas for potential improvement through an examination of the output during the development process.

## Results

The results for the quantitative and qualitative performance analysis both for NER and topic modelling task is given in this section.

### Quantitative analysis

#### Evaluating named entity recognition module

We use the benchmark NER datasets: NCBI-Disease [67], i2b2-clinical [68], and i2b2 2012 [69] for evaluation. For preparing our own test set, we performed the following steps: (i) we use the pre-trained BERT-based NER models (ner_jsl_biobert and bert-clinical for adverse drug events) that are fine-tuned on a dataset of annotated text containing mentions of drugs, clinical entities (disease, symptoms etc.), and demographics (age, gender, race); (ii) to annotate an unlabelled corpus, we first select a sample of 1,500 tweets, pre-process the text, and input it into the pre-trained models. These models then generate labels and start/end positions for each named entity in the text. This approach can be considered as an active learning process [70], where we use pre-trained models to annotate, re-annotate, and enhance the quality of our test set. The details of benchmark datasets, baselines, and training platform are given in Appendix A, Table S3. The overall NER performance using different datasets over baselines is given in Table 1.

Table 1 presents the performance of various models on different datasets, showcasing the mean and standard deviation (± SD) of precision (P), recall (R), and F_1_-score (F1) metrics across fivefold cross-validation. The comparison of various models reveals that BioBERT consistently achieves the highest F_1_-scores across most datasets. BLUE also performs as next-best to BioBERT. Our proposed approach demonstrates better performance on the custom test set and competitive performance on other datasets. BiLSTM-CRF and Att-BiLSTM-CRF show good performance, but they are outperformed by BERT-like methods. Then comes the performance of CollabNet after BiLSTM-CRF and Att-BiLSTM-CRF.

We also observe in Table 1 that our proposed approach outperforms all other methods in terms of F_1_-score on all test sets, except for i2b2–2012, where BioBERT outperforms our approach by a marginal difference of ~ 0.1%. The overall performance of BERT-based methods (BioBERT and BLUE) is better than the BiLSTM-based methods (BiLSTM-CRF, CollabNet, and Att-BiLSTM). This result perhaps indicates that pre-trained BERT-like models offer better contextualized representations of the data. However, there is little performance difference between two sets of methods, suggesting that simple models can be used if resource utilization is a concern (BERT-like methods are resource-consuming).

Our hybrid approach for NER combines the benefits of the optimized BioBERT model with the performance boost provided by the traditional BiLSTM models. This combination leads to an overall improvement in performance.

#### Evaluating topic modelling module

**Fig. 3:**
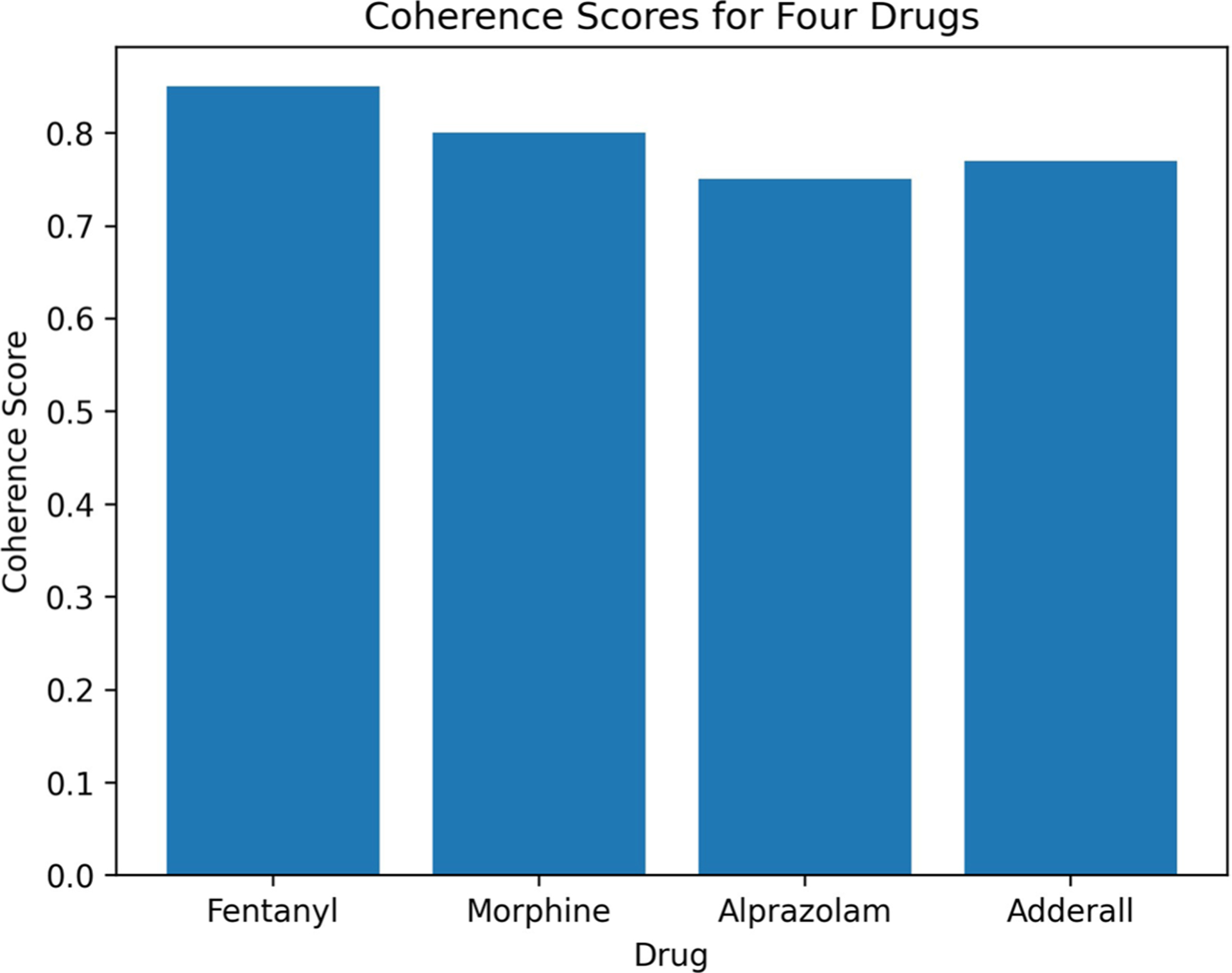
The coherence scores for the topics generated by BERTopic for four drugs: Fentanyl, Morphine, Alprazolam, and Adderall^®^. The coherence scores range between 0–1, with values closer to 1 indicating high coherence among the topics

The topic modelling that we used in this work is an unsupervised task, which means there are no gold labels to compare the model performance. In order to evaluate the topic modelling task on our test set, we employ the coherence score metric [75], which is a measure of how well the topics generated by a topic model are related to each other. BERTopic [61] uses c-TF-IDF to identify the most important words within each topic and then calculates the coherence score based on the cosine similarity between the word vectors of these important words. In Fig. 3, we evaluate the coherence of topics generated by the topic modelling task for four drugs: Fentanyl, Morphine, Alprazolam, and Adderall^®^.

Overall, we observe in Fig. 3 that Fentanyl, Alprazolam, Morphine and Adderall have coherence scores of between 0.75 – 0.85 indicating that the topics generated by the model are highly related and semantically coherent. Alprazolam has a slightly lower coherence score of 0.75 but it still indicates high coherence among the topics. The results suggest that the topic modelling approach used is effective in generating coherent topics related to each drug. The performance of this approach can be attributed to the successful integration and functioning of the predecessor components in the framework, which facilitate the accurate identification and representation of relevant information from the input data.

These results can be useful in understanding the most common and important themes discussed on social media related to these drugs. Nevertheless, these results are based on an unsupervised approach and may not capture all relevant topics related to these drugs. Further analysis and validation may be necessary to fully understand the topics related to these drugs in social media.

### Qualitative analysis

Qualitative analysis involves examining patterns and insights in text data to gain a better understanding of non-medical prescription drug use.

#### Analysis of the named entities

**Fig. 4:**
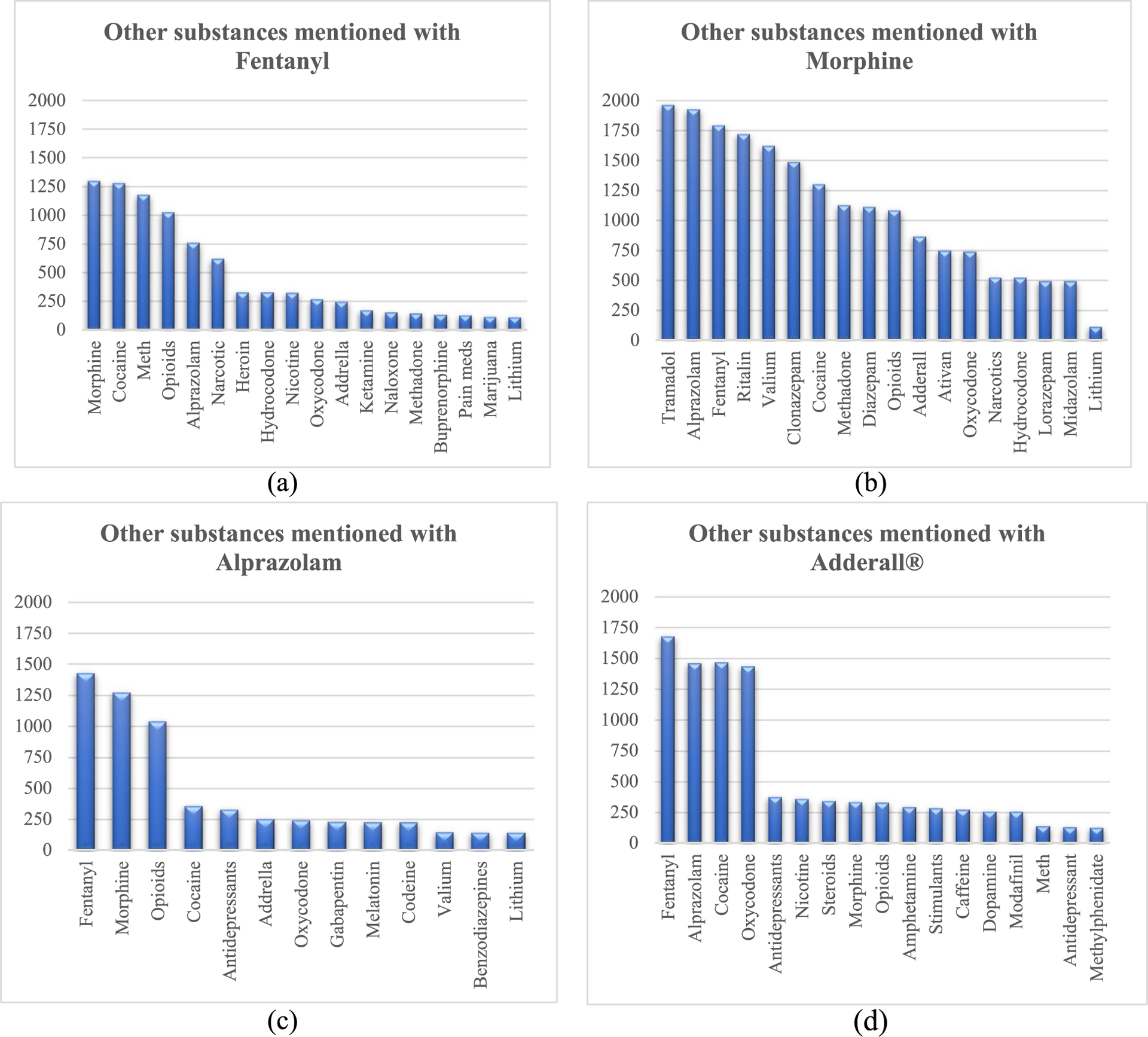
Most frequent substances mentioned in tweets along with (**a**) fentanyl, (**b**) morphine, (**c**) alprazolam, and (**d**) Adderall^®^

In Fig. 4, we display the names of other substances that were mentioned alongside fentanyl, morphine, Adderall^®^, and alprazolam. This information can be useful in identifying substances that are commonly co-used with these four substances.

The results presented in Fig. 4 show a connection between the mention of morphine and other opioid pain medications such as fentanyl and tramadol. In addition, fentanyl is often mentioned alongside alprazolam and Adderall^®^. These patterns suggest possible associations or co-usage among these substances, which can be valuable information for further research on this topic. In Appendix A: Figure S1, we also provide a comprehensive list of the most frequently mentioned substances in tweets, offering additional insights for studying substance use and their potential relationships.

**Fig. 5:**
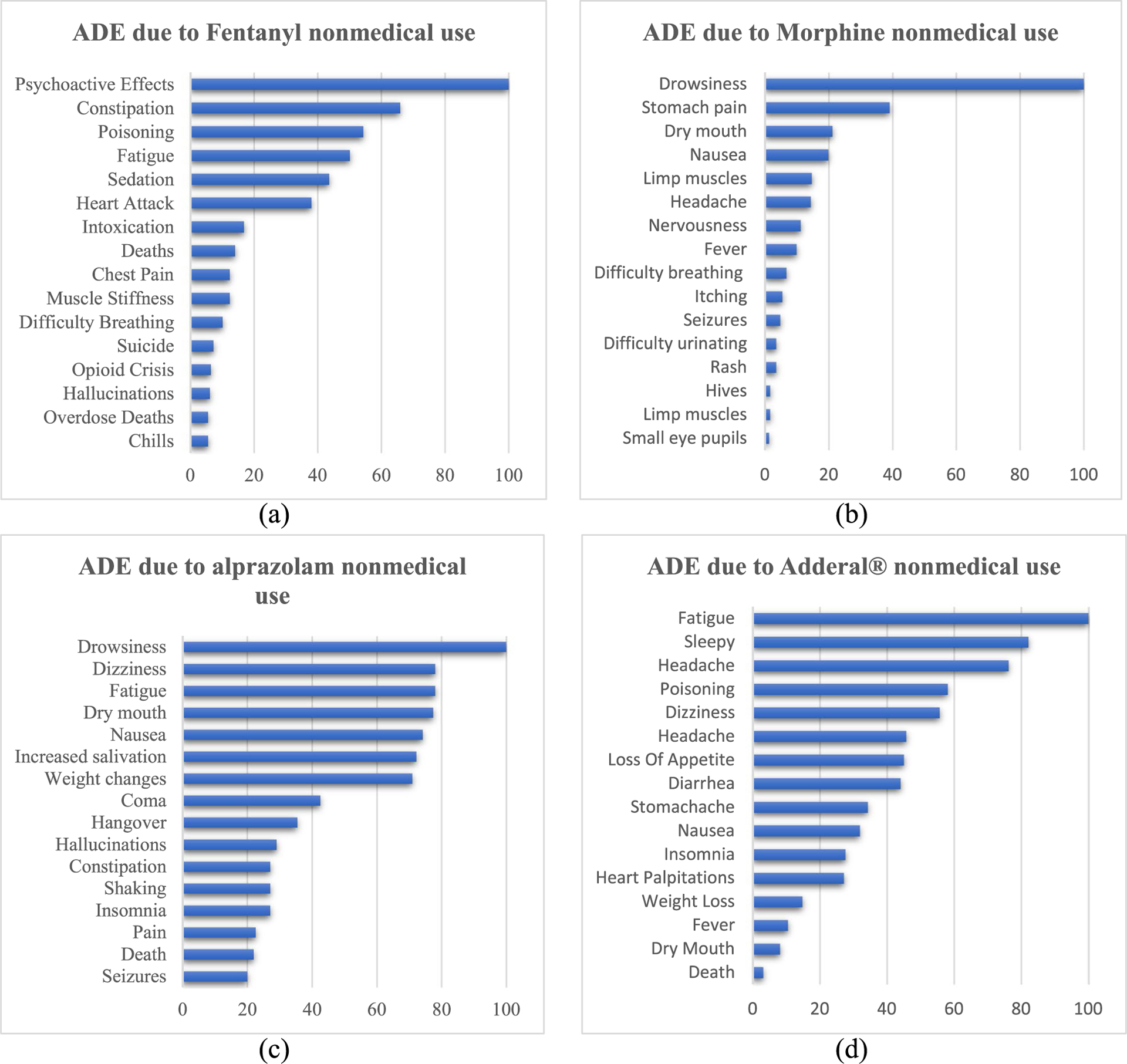
Percentage distribution of adverse drug events (ADE) due to substance non-medical use. The scores are normalized, x-axis presents the frequency distribution and y-axis presents the ADE

Adverse Drug Events (ADE) [76] are harmful or undesirable effects that result from the use of medications, which can include medical, psychological, or non-medical outcomes. Considering this definition, we present the distribution of ADEs, shown in Fig. 5, extracted from the chatter concerning various medications.

The results in Fig. 5 show that the most frequently reported ADEs are drowsiness, fatigue, and pain, highlighting potential concerns for consumers of these medications. Additionally, common side effects such as sickness, hallucinations, nausea, headaches, dizziness, drowsiness, fatigue are also mentioned in tweets, indicating potential connections between the medications and these effects. To further explore the impact of these medications, the frequency distribution of psychological symptoms associated with the non-medical use of these substances is provided in Appendix A: Figure S2. An example of the ADE annotation process using a sample tweet can be found in Appendix A: Figure S3.

Table 2 presents the treatment options suggested in tweets for non-medical use of substances, including fentanyl, alprazolam, Adderall^®^, and morphine.

We observe in Table 2 that the most recommended treatments for these four substances are rehab, group therapy, and cognitive-behavioral therapy. In some cases, the specific treatments suggested for each substance may differ. For example, immunotherapy is suggested for fentanyl due to its potential risks and addictive nature as a potent opioid. We also observe that calming exercises and nature therapy are suggested as alternatives to alprazolam, a medication used to treat anxiety disorders, to promote relaxation and stress reduction. These recommendations in tweets are probably not evidence-based, and more research is required to determine the efficacy and safety of these treatments for non-medical substance use.

We also show some demographic analysis based on age and gender in Appendix A S5. The age and gender entities are obtained using our NER model from the dataset. Overall, the results (Appendix A: Figure S4) indicate that both males and females report non-medical use of these substances, with over 55% of users being male. We also find (Appendix A: Figure S5), that the majority of non-medical substance use reports on Twitter come from people aged 25 to 40, who also make up the largest demographic of Twitter users [77]. These figures are based on mentions of age and drug use in tweets and may not represent the whole population.

#### Analysis of topic modelling results

The outputs of topic modelling are shown in Table 3 and discussed next.

As seen in Table 3, the topic-words related to these substances are associated with its their medical and non-medical use, misuse, and impact on individuals and society. The findings show that the Fentanyl is a synthetic opioid that is used to treat severe pain and it is a highly potent drug. It can cause addiction, severe overdose, and other serious side effects. Alprazolam is a medication used to treat anxiety, panic disorder, and phobias, but it has a potential for abuse, addiction, and serious side effects. Adderall^®^ is a stimulant medication prescribed to treat ADHD, it can help with attention, focus, and impulse control but its use may also have negative impacts on mental health and lead to addiction or substance use disorder. Morphine is a powerful opioid pain medication. It also can cause physical side effects such as muscle aches, agitation, withdrawal symptoms and insomnia. It is used in the hospital setting for pain management and reduction. It is also used for pain management of terminal illnesses and in older population. All these medications have side effects; therefore, they should only be taken under medical supervision and with a proper evaluation of the benefits vs risks, but as noted have a high potential for non-medical use and addiction.

**Fig. 6:**
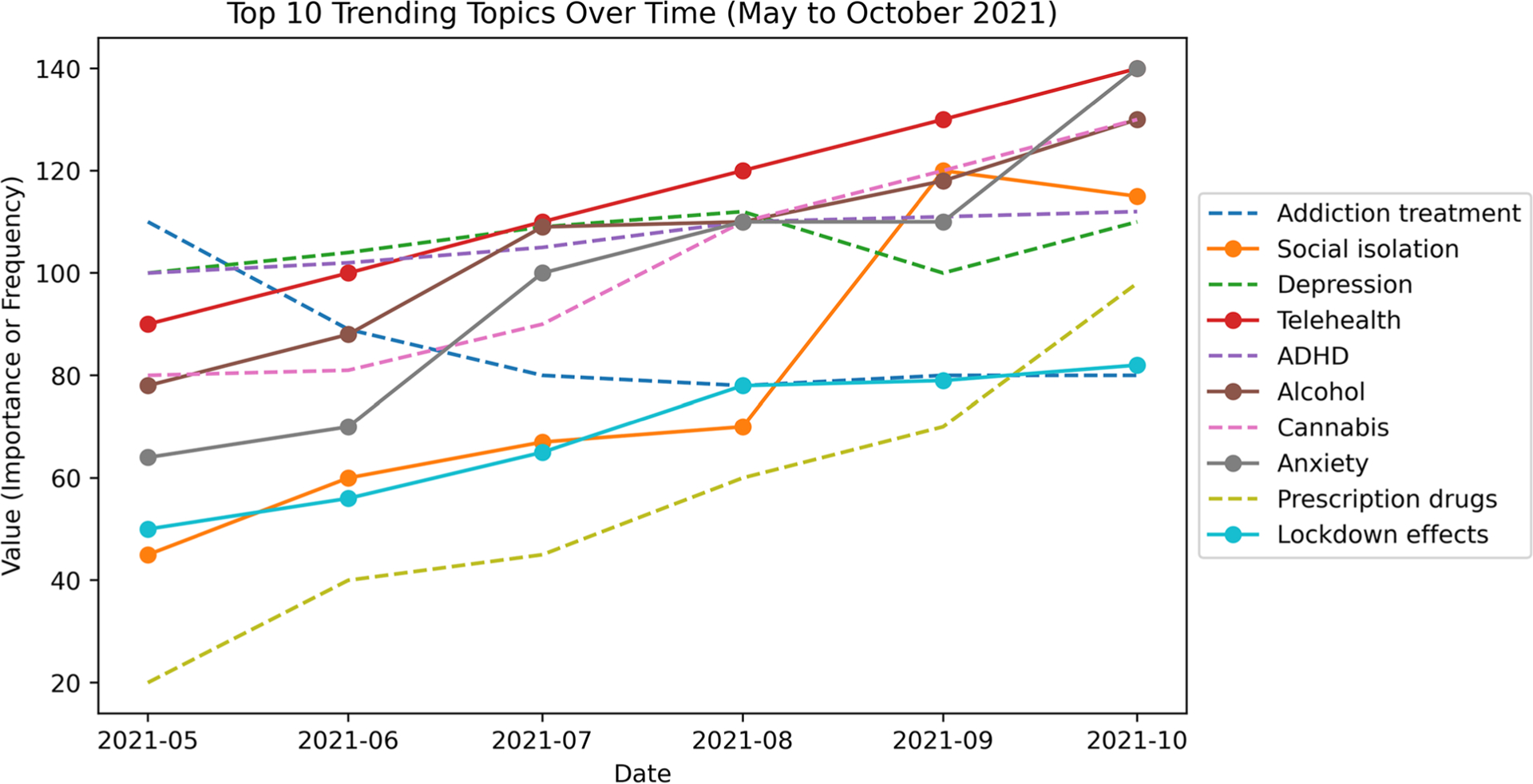
Top-10 Substance Use Topics from May 2021 to October 2021. Each line or marker represents a specific topic, with the x-axis showing the date and the y-axis indicating the value (importance or frequency) of each topic

Next, we show the distribution of significant topics over time based on their frequency in Fig. 6. This plot illustrates the changes in frequency of various topics over time, providing insights into the relative popularity and interest in these topics during the specified period.

We observe in Fig. 6 that certain topics, such as addiction treatment and prescription drugs maintain relatively stable frequency values throughout the period. Topics like social isolation and lockdown effects display moderate levels of interest with slight fluctuations in frequency values. Other topics such as depression and anxiety remain popular topics, and telehealth topic show a gradual increase in frequency values over time. The significance of the telehealth and anxiety topics could be attributed to the COVID-19 pandemic [26], during which lockdown measures were implemented, leading to heightened depression as a consequence of the restrictions imposed and increased interest in telehealth. The analysis also reveals varying levels of interest in topics like ADHD, alcohol, cannabis, anxiety, and the effects of lockdown, with fluctuating frequency values suggesting changing levels of engagement and discussions around these subjects. Further analysis of topics is presented in Appendix A: Figure S6 and Appendix A: Figure S7.

## Discussion

### Principal results

In this paper, we describe our NLP framework for identifying multi-faceted information from Twitter chatter regarding substance non-medical use. The framework is integrated with our existing end-to-end pipeline that focuses on collecting Twitter chatter, identifying posts that represent potential non-medical use, and characterizing the chatter in terms of demographics. Our framework involves a combination of supervised NER methods and unsupervised topic modelling. The results show that our NLP framework for content analysis can reveal multi-faceted information specific to each substance including but not limited to substances/medications (that are potentially co-used with our target substances), ADEs, symptoms, and therapies.

In our experiments for this paper, we included three different categories of substances—opioids, stimulants and benzodiazepines, and the chatter analyses revealed the differing contents associated with chatter for each category. The inclusion of two opioids was also intentional, as our analyses show subtle differences between the two substances belonging to this class. The analyses of the outputs revealed the existence of ADEs, psychological effects, and therapies. We find that although these drugs may be primarily used to treat pain or certain disorders (e.g., fentanyl [15], which is used to treat cancer patients’ pain), there is much misuse of these substances among people. Our analyses also show that when integrated with the bigger pipeline, the analyses can shed light on information revealed by targeted demographics (e.g., different age groups).

### Practical implications

The proposed NLP framework can be used by healthcare and public health stakeholders to study substance use and the symptoms, ADEs, effects, treatments, and trends related to specific substance use. One can use this pipeline with little or no code change on a new set of medications and on different data to provide timely analysis of the data and guide the prevention and self-harm reduction efforts. A key advantage of a social media-based framework is that the information can potentially be collected in close to real-time, and thus, the typical lag associated with more traditional sources of surveillance can be overcome. While we do not envision that this framework will replace traditional surveillance systems, such as overdose monitoring systems and surveys, it can complement them. It could also flag a potentially serious or lethal situation, such as when particularly potent fentanyl may be leading to more frequent or more serious over- doses as noted in social media. It could alert healthcare providers and even law enforcement to be prepared and respond.

### Limitations

This study has some limitations, which are discussed below:

*Time-span*: The dataset used in this study only covers a relatively short time span of five months (May 2021 to October 2021). To better understand the system’s performance and its ability to adapt to evolving trends, it would be beneficial to examine the results within a larger and/or more recent time window. Previous studies [78, 79] have shown that deep neural and transformer-based models can infer patterns even from relatively small datasets and are generalizable to larger datasets. Future research should address this limitation by incorporating an expanded dataset that encompasses a wider range of temporal variations, allowing for a more robust evaluation of the proposed framework’s capabilities.
*Causality*: This framework does not explicitly mention causality in the relationships between the drugs and their effects. This can make it challenging to determine whether a drug is being taken to alleviate a symptom or if a symptom is being caused by the drug. More research is needed to delve into the causal aspects of these relationships.
*Reliability of social media data*: Social media is not, generally, considered a trustworthy source of health information [80] and thus the veracity of the data gathered from social media discussions does not reflect the evidence or viewpoint from public health experts [81]. It is important to acknowledge this limitation when interpreting and generalizing the findings obtained from social media data.

### Future research directions

We propose additional directions for future research. One direction is to explore the utilization of transfer learning techniques to obtain more specific embeddings for NER substance/drug identification tasks. Transfer learning can leverage foundation models and knowledge from related domains to enhance the performance of the NER task, enabling better identification and classification of named entities.

Further, creating a knowledge graph [82] can greatly contribute to the integration and connectivity of the extracted entities and topics. By building a knowledge graph, it becomes possible to establish relationships and connections between different entities and their networks. This approach can also enrich the NER task [22] and topic modelling with external knowledge bases, fostering a more adaptable and comprehensive understanding of the data.

It would be good to investigate more techniques to incorporate contextual information [83], such as temporal information or user demographics, could provide further insights into the dynamics of the identified entities and topics. Analysing how these factors influence the occurrence and distribution of named entities and topics can contribute to a better understanding of the underlying patterns and trends.

It is worth considering the inclusion of social determinants of health [84] in future research, particularly focusing on aspects such as health equity and resource distribution. Understanding how these social factors influence health outcomes and the availability of resources for treatments of those affected by drug additions or substance misuse can provide valuable insights into disparities and inequities within populations.

## Conclusion

This study proposes an NLP framework to analyse Twitter chatter about the non-medical use of prescription medications. The framework uses the NER method to identify posts discussing non-medical use, and then applies topic modelling to uncover latent topics related to the use of these medications. We test the framework with four medications (morphine, fentanyl, Adderall^®^, and alprazolam).The detailed analysis provides insights into the use, non-medical use, and consequences of these medications in users. The framework developed in this research can be applied to similar discussions on other substances, making it a valuable tool for studying substance use and related behaviours on social media. Further research can validate the accuracy and predictive value of this information on the prevention, assessment, and management of these disorders.

## Supplementary Material

supplementary material

## Figures and Tables

**Table 1:** Performance evaluation of the NER task uses Precision (P), Recall (R), and F1-score (F1). Bold indicates the best score. All baselines are tuned to their optimal settings, and the best result for each method is reported. The train-test split information for each dataset follows the original papers. For our test data, a standard ratio of 70–15–15 is employed. A fivefold cross-validation is conducted, providing mean and standard deviation (SD) (Mean ± SD) values for each measure

Model/Dataset		NCBI [67]	i2b2-clinical [68]	i2b2-2012 [69]	Our test set
BiLSTM-CRF [71]	**P**	88.3 ± 1.9	89.2 ± 1.6	87.4 ± 2.2	90.1 ± 2.1
	**R**	90.1 ± 1.7	91.1 ± 1.8	89.1 ± 2.0	92.1 ± 1.9
	**F1**	89.2 ± 1.8	90.1 ± 1.7	88.2 ± 2.1	91.1 ± 2.0
Att-BiLSTM-CRF [72]	**P**	85.5 ± 1.5	88.6 ± 1.7	89.8 ± 1.9	88.1 ± 1.6
	**R**	86.2 ± 1.4	90.0 ± 1.8	92.0 ± 1.7	92.2 ± 1.5
	**F1**	85.9 ± 1.4	89.3 ± 1.7	90.9 ± 1.8	90.1 ± 1.5
CollabNet [73]	**P**	82.1 ± 1.2	84.2 ± 1.3	83.1 ± 1.4	86.7 ± 1.5
	**R**	84.1 ± 1.3	85.4 ± 1.2	84.3 ± 1.3	88.1 ± 1.4
	**F1**	83.1 ± 1.2	84.8 ± 1.2	83.7 ± 1.3	87.4 ± 1.4
BLUE [74]	**P**	90.4 ± 1.7	92.1 ± 1.5	91.5 ± 1.6	91.0 ± 1.4
	**R**	92.9 ± 1.6	93.2 ± 1.4	92.1 ± 1.5	93.0 ± 1.3
	**F1**	91.6 ± 1.6	92.6 ± 1.4	91.8 ± 1.5	91.9 ± 1.3
BioBERT [45]	**P**	91.2 ± 1.8	91.0 ± 1.7	91.4 ± 1.9	92.3 ± 1.6
	**R**	92.3 ± 1.7	91.1 ± 1.8	**93.1 ± 1.7**	93.1 ± 1.5
	**F1**	91.7 ± 1.7	91.0 ± 1.7	**92.3 ± 1.8**	92.7 ± 1.5
Our approach	**P**	**91.9 ± 1.6**	**92.1 ± 1.4**	**92.2 ± 1.5**	**94.8 ± 1.3**
	**R**	**93.4 ± 1.5**	**93.3 ± 1.3**	92.1 ± 1.4	**95.1 ± 1.2**
	**F1**	**92.6 ± 1.5**	**92.7 ± 1.3**	92.2 ± 1.4	**94.9 ± 1.2**

**Table 2:** Treatments recommended in tweets for non-medical substance use are derived from identified named entities, considering those with frequencies of occurrence of over 70%

Substance	Treatment Options	Examples of Tweeted Suggestions
Fentanyl	Immunotherapy, Rehab, Radiation, Individual Therapy, Psychedelic-Assisted Therapy, Coercive Treatment, Detox, Group Therapy, Cognitive Behaviour Therapy, Medication	“Rehab is a great option for fentanyl addiction recovery”, “Individual therapy helped me cope with fentanyl withdrawal symptoms”, “Psychedelic-assisted therapy shows promise for treating fentanyl addiction”
Alprazolam	Rehab, Group Therapy, Medication, Yoga, Counseling, Family Therapy, Motivational Therapy, Emotional Behavioural Therapy, Nature Therapy	“Counseling helped me overcome anxiety without relying on alprazolam”, “Nature therapy provides a calming alternative to alprazolam”, “I suggest that Alprazolam users should consider group therapy for support”
Adderall^®^	Rehab, Cognitive Behavioural Therapy, Trauma Therapy, Behavioral Therapy, Calming Exercises, Regenerative Therapy, Psychedelic-Assisted Therapy, Medical Supervision, Mindfulness Meditation, Aerobic Exercise Training, Breathing Treatments	“Trauma therapy is useful to address underlying issues leading to Adderall abuse”, “We suggest mindfulness meditation to reduce stress and boost focus without Adderall”, “Aerobic exercise training can boost energy levels without Adderall”,
Morphine	Rehab, Group Therapy, Exposure Therapy, Distraction Therapy, Mindfulness Meditation, Cognitive Behavioural Therapy, Aerobic Exercise Training, Breathing Treatments	“For morphine addition, group therapy is a safe therapy”, “Exposure therapy can help in overcoming fears associated with morphine use”, “Aerobic exercise training is useful to increase natural endorphins and reduce reliance on morphine”

**Table 3:** Topic-words, the top-5 words in each topic associated with each substance

Substance	Topic-words	Topic
Fentanyl	Terrible, wild, depressed, therapist, crying	Emotional states
	Prescription, doctor, therapy, pain, management	Medical use
	Drugs, cocaine, mental, children, methamphetamine	Non-medical use
	Behavioral, prevention, therapy, environment, support	Treatment
	Withdrawal, overdose, tolerance, misuse, dependence	Side effects, complications
Alprazolam	Anxiety, panic, phobia, stress, nervousness	Anxiety and panic disorders
	Prescription, dosage, side effects, addiction, abuse	Medication and non-medical use
	Depression, insomnia, mood swings, cognitive, anxiety	Mental health
	Therapy, benzodiazepines, management, doctor, brain	Treatment
	Adults, women, pregnant, children, adolescents	Effects on specific populations
Adderall^®^	Concentration, hyperactivity, attention, ability, forget	ADHD
	Focus, exam, energy, motivation, productivity	Students
	Anxiety, insomnia, mood, agitation, nervousness	Mental health
	Overuse, cardiovascular, addiction, withdrawal, dependence	Side effects, complications
	Stimulation, dopamine, nervous, neuro, synapse	Neurological effects
Morphine	Pain, management, reduction, hospital, drug	Pain management medical use
	Drowsiness, addition, nausea, breathing, confusion	Side effects, complications
	Sedative, antidepressant, medication, prescription, interaction	Interaction with other medicine
	Agitation, muscle, ache, withdrawal, insomnia	Withdrawal symptoms
	Pain, terminal, old, end, illness	Palliative care medical use

## Data Availability

The code for the NLP framework and the IDs for the tweets used in this study will be made available upon request after the publication of this manuscript. Please contact S.R. for code (shaina.raza@utoronto.ca). Please contact A.S. (abeed@dbmi.emory.edu) or S.L. (sahithi.krishnaveni.lakamana@emory.edu) for the data.

## Abbreviations

NLP: Natural Language Processing
API: Application Programming Interface
ADE: Adverse Drug Event
NER: Named Entity Recognition
SAMHSA: Substance Abuse and Mental Health Services Administration
CDC: Centers for Disease Control and Prevention
FDA: Food and Drug Administration
ADHD: Attention Deficit Hyperactivity Disorder
BERT: Bidirectional Encoder Representations from Transformers
BiLSTM: Bidirectional Long Short-Term Memory
CNN: Convolutional Neural Networks
CRF: Conditional Random Field
IOB: Inside, Outside, Before
UMAP: Uniform Manifold Approximation and Projection
HDBSCAN: Hierarchical and Density Based Clustering
c-TFI-DF: Class-based Term Frequency-Inverse Document Frequency
