# Supplementary material for: A framework for multi-faceted content analysis of social media chatter regarding non-medical use of prescription medications
Source: BMC Digit Health. Author manuscript; Available in PMC 2023 Sep 7. (PMC10483682; doi:10.1186/s44247-023-00029-w)
Supplement: supplementary material [file NIHMS1926102-supplement-supplementary_material.docx]

**Research article**

**Title: A framework for multi-faceted content analysis of social media chatter regarding non-medical use of prescription medications**

**Shaina Raza**, PhD, Dalla Lana School of Public Health, University of Toronto, Toronto, ON, Canada; Vector Institute for Artificial Intelligence, Toronto, ON, Canada.

**Brian Schwartz**, MD, MScCH, Dalla Lana School of Public Health, University of Toronto, Toronto, ON, Canada.

**Sahithi Lakamana**, MS, Department of Biomedical Informatics, School of Medicine, Emory University, Atlanta, GA, USA

**Yao Ge**, MS, Department of Biomedical Informatics, School of Medicine, Emory University, Atlanta, GA, USA

**Abeed Sarker**, PhD, Department of Biomedical Informatics, School of Medicine, Emory University, Atlanta, GA, USA

**Corresponding author:** PhD, Dalla Lana School of Public Health, University of Toronto, Toronto, ON, Canada; Vector Institute for Artificial Intelligence, , Toronto, ON, Canada [shaina.raza@utoronto.ca](mailto:shaina.raza@utoronto.ca)

**Appendix A**

**Table S1**: Regular expressions used

**Table S2.** Named entities used

**Table S3**. Benchmark datasets, baseline methods used and training platform

**Figure S1**. Most frequent drug names mentioned in tweets. These results are calculated by taking the chunks of named entities and calculating their frequencies and finding the trends.

**Figure S2**: Distribution of psychological symptoms mentioned in the tweets. The scores are normalized, x-axis presents the frequency distribution and y-axis presents the condition.

**Figure S3**: Adverse drug event on a sample tweet.

**Figure S4**: Percentage of Male and Female reported with non-medical use of substance.

**Figure S5:** Non-medical substance usage among different age groups.

**Figure S6**: Hierarchical topics.

**Figure S7**: Similarity matrix of topics.

**Table S1**. Regular expressions used.

| **Regular expression**  r'(?<!\w\.\w.)(?<![A-Z][a-z]\.)(?<=\.\|\?\|\!)\s  (?<!\w\.\w.): it asserts that the match is not preceded by a word character (\w), a period (\.), another word character, and any character.  (?<![A-Z][a-z]\.): it asserts that the match is not preceded by an uppercase letter ([A-Z]), a lowercase letter ([a-z]), and a period.  (?<=\.\|\?\|\!): it asserts that the match is preceded by a period (\.), a question mark (\?), or an exclamation point (\!).  **Example**  import re  class PreProcessor:  def __init__(self, text):  self.text = text  def remove_noise(self):  # Remove extra spaces, tabs, and newlines  self.text = re.sub(r'\s+', ' ', self.text).strip()  def sentence_tokenize(self):  # Regular expression for sentence boundaries  sentence_boundary_regex = r'(?<!\w\.\w.)(?<![A-Z][a-z]\.)(?<=\.\|\?\|\!)\s'  sentences = re.split(sentence_boundary_regex, self.text)  return sentences  def preprocess(self):  self.remove_noise()  sentences = self.sentence_tokenize()  return sentences  # Example text  text = "The Parkinson disease affects the central nervous system (CNS) and is caused by the loss of dopamine-producing neurons. Some studies suggest that the prevalence of Parkinson’s disease is 1% in people aged 60 and older. Can early intervention help? Yes, early intervention and treatment may slow the disease's progression."  # Preprocess the text  preprocessor = PreProcessor(text)  preprocessed_text = preprocessor.preprocess()  # Print the preprocessed text  for i, sentence in enumerate(preprocessed_text):  print(f"Sentence {i + 1}: {sentence.strip()}")  **Output**  Sentence 1: The Parkinson disease affects the central nervous system (CNS) and is caused by the loss of dopamine-producing neurons.  Sentence 2: Some studies suggest that the prevalence of Parkinson’s disease is 1% in people aged 60 and older.  Sentence 3: Can early intervention help?  Sentence 4: Yes, early intervention and treatment may slow the disease's progression. |
| --- |
|  |

**Table S2.** Named entities used

| **Pre-trained Model:** ner_jsl_biobert [1]  **Named entities**: Strength, Pregnancy_Delivery_Puerperium, Female_Reproductive_Status, Fetus_NewBorn, Age, Alcohol, Treatment, Internal_organ_or_component, Vital_Signs_Header, Dosage, Employment, Gender, Disease_Syndrome_Disorder, Pregnancy, Symptom, Clinical_Dept, Medical_Device, Temperature, Hypertension, Cerebrovascular_Disease, Psychological_Condition, Respiration, Direction, Metastasis, Injury_or_Poisoning, Birth_Entity, Allergen, Labour_Delivery, Overweight, Family_History_Header, Section_Header, Diabetes, Hyperlipidemia, Death_Entity, Route, Duration, Admission_Discharge, Total_Cholesterol, Performance_Status, LDL, RelativeDate, Test_Result, Height, Procedure, Date, Cancer_Modifier, BMI, External_body_part_or_region, Kidney_Disease, Modifier, Oncology_Therapy, Drug_BrandName, Form, Substance, Social_History_Header, Obesity, Oncological, Sexually_Active_or_Sexual_Orientation, EKG_Findings, Oxygen_Therapy, Frequency, Relationship_Status, Communicable_Disease, Imaging_Technique, Vaccine, Pulse, Tumor_Finding, Heart_Disease, Time, ImagingFindings, HDL, O2_Saturation, Weight, Medical_History_Header, Blood_Pressure, Puerperium, Smoking, Substance_Quantity, RelativeTime, Test, Race_Ethnicity, Diet, Staging, Triglycerides, Drug_Ingredient, VS_Finding. |
| --- |
| **Pre-trained Model:** bert-clinical for adverse drug events [1].  **Named entities:** ADE DRUG, ADE. |

**Table S3**. Benchmark datasets, baseline methods used and training platform

| **Benchmark Datasets** | | |
| --- | --- | --- |
|  | **Entities** | **Corpus** |
| NCBI-Disease [2] | Diseases | 793 PubMed abstracts |
| i2b2-Clinical [3] | Problem, Treatment, and Test. | 426 discharge summaries |
| I2b2 2012 [4] | Clinical (problems, tests, treatments, clinical departments, occurrences (admission, discharge) and evidence). | 310 discharge summaries |
| **Baseline methods used** | | |
| 1. BiLSTM-CRF [5], Bidirectional LSTMs and CRF architecture for NER. 2. Att-BiLSTM-CRF [6], an attention (Att) based BiLSTM model with a CRF layer for chemical NER task. 3. CollabNet [7], which is a collaboration of deep neural networks, i.e., BiLSTM-CRF and with a single task model trained for each specific entity type. 4. BLUE [8] , biomedical language understanding evaluation (BLUE) with BERT based pre-training for the biomedical language representation tasks. 5. BioBERT [9], a pre-trained biomedical language representation model for biomedical text mining. We use BioBERT-Base v1.2. | | |
| **Training environment** | | |
| We employ the commonly used metrics: precision, recall, F1-score, in the field of NER research[10] to evaluate the performance NER task. We set the experimental environment as: Intel(R) Core(TM) i7-8565U CPU @ 1.80GHz, 1.99 GHz, 16.0 GB RAM, 64-bit operating system, x64-based processor; GPU: Google Colab Pro with cloud-based GPUs (K80, P100, or T4), 32GB RAM and training capabilities. We connect the Google Colab to Google Drive to get enough storage for transfer learning. | | |

**Figure S1**. Most frequent substances mentioned in tweets. These results are calculated by taking the chunks of named entities and calculating their frequencies and finding the trends.

**Figure S2**: Distribution of psychological symptoms mentioned in the tweets. The scores are normalized, x-axis presents the frequency distribution and y-axis presents the condition.

| (a) | (b) |
| --- | --- |
| (c) | (d) |

According to these findings, 'anxiety' is the most common psychological condition. Other conditions mentioned are mood disorders, panic attacks, ADHD (attention-deficit/hyperactivity disorder), PTSD (post-traumatic stress disorder), among other psychological conditions. SAD (seasonal affective disorder), SUD (substance use disorder), ADD (attention deficit disorder), bipolar and hysteria.

**Figure S3**: Adverse drug event on a sample tweet.


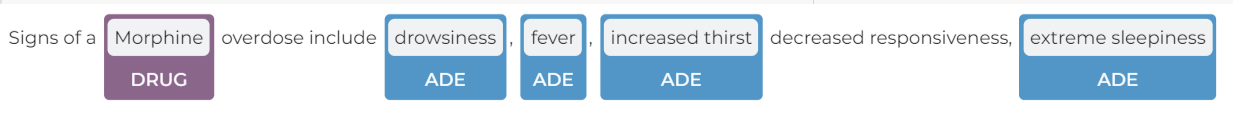


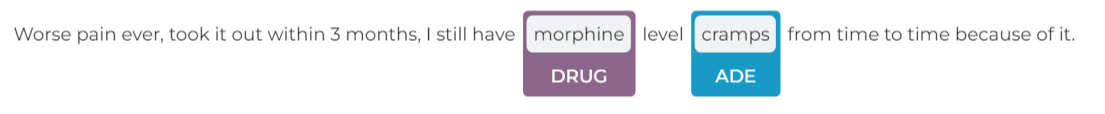


**Figure S4**: Percentage of Male and Female reported with non-medical use of substance.

**Figure S5:** Nonmedical substance usage among different age groups.

**Figure S6**: Hierarchical topics.


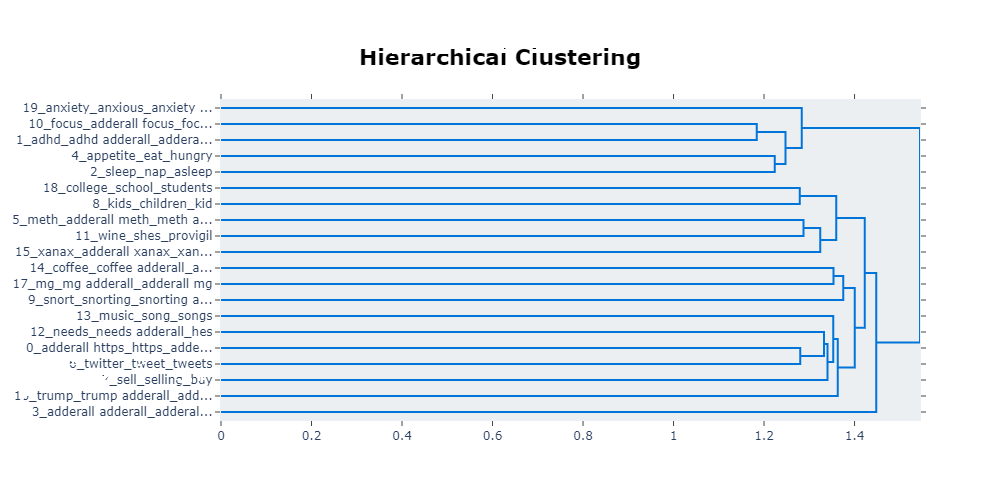


By looking at the first level (level 0) of the dendrogram, we can see that topics with the same words have been grouped together. For example, Topic 19 (anxiety) and topic 1 (adderall) have been grouped together because of their closeness.

**Figure S7**: Similarity matrix of topics.


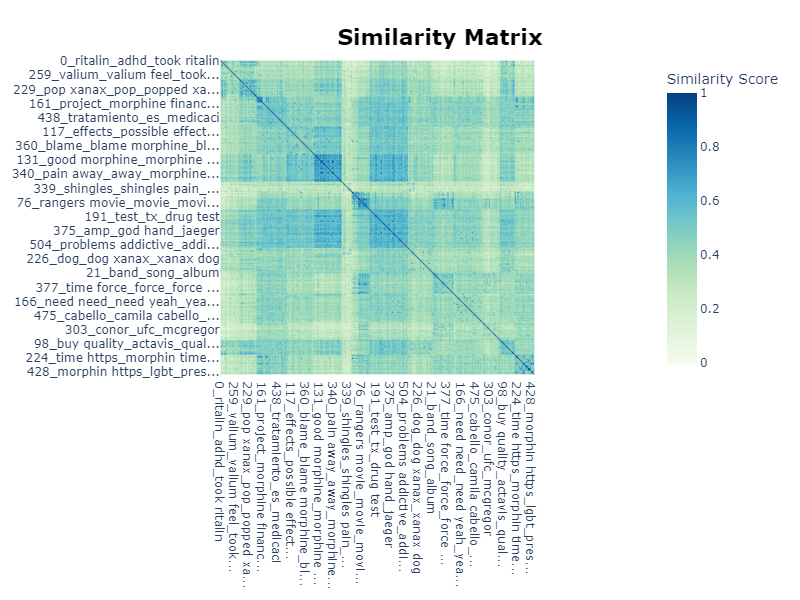


The similarity matrix is calculated by simply applying cosine similarities through the topic embeddings. This matrix indicates how similar certain topics are to each other. A score closer to 0 indicates more similarity and far from 0 show that topics are dissimilar. That is why, similar topics have a score of 0 , for example topic 428 and 428 on both axes.

**References**

1. John Snow Labs. John Snow Labs - Spark NLP. 2022.

2. Doğan RI, Leaman R, Lu Z. NCBI disease corpus: A resource for disease name recognition and concept normalization. J Biomed Inform. 2014;47:1–10.

3. Uzuner Ö, South BR, Shen S, DuVall SL. 2010 i2b2/VA challenge on concepts, assertions, and relations in clinical text. J Am Med Informatics Assoc. 2011;18:552–6.

4. Sun W, Rumshisky A, Uzuner O. Evaluating temporal relations in clinical text: 2012 i2b2 challenge. J Am Med Informatics Assoc. 2013;20:806–13.

5. Lample G, Ballesteros M, Subramanian S, Kawakami K, Dyer C. Neural architectures for named entity recognition. arXiv Prepr arXiv160301360. 2016.

6. Luo L, Yang Z, Yang P, Zhang Y, Wang L, Lin H, et al. An attention-based BiLSTM-CRF approach to document-level chemical named entity recognition. Bioinformatics. 2018;34:1381–8.

7. Yoon W, So CH, Lee J, Kang J. Collabonet: collaboration of deep neural networks for biomedical named entity recognition. BMC Bioinformatics. 2019;20:55–65.

8. Peng Y, Yan S, Lu Z. Transfer learning in biomedical natural language processing: an evaluation of BERT and ELMo on ten benchmarking datasets. arXiv Prepr arXiv190605474. 2019.

9. Lee J, Yoon W, Kim S, Kim D, Kim S, So CH, et al. BioBERT: A pre-trained biomedical language representation model for biomedical text mining. Bioinformatics. 2020;36:1234–40.

10. Cho H, Lee H. Biomedical named entity recognition using deep neural networks with contextual information. BMC Bioinformatics. 2019;20:1–11.
